# Mining the Royal Jelly Proteins: Combinatorial Hexapeptide Ligand Library Significantly Improves the MS-Based Proteomic Identification in Complex Biological Samples

**DOI:** 10.3390/molecules26092762

**Published:** 2021-05-07

**Authors:** Eliza Matuszewska, Joanna Matysiak, Grzegorz Rosiński, Elżbieta Kędzia, Weronika Ząbek, Jarosław Zawadziński, Jan Matysiak

**Affiliations:** 1 Department of Inorganic and Analytical Chemistry, Poznan University of Medical Sciences, Grunwaldzka 6 Street, 60-780 Poznań, Poland; eliza.matuszewska@ump.edu.pl; 2 Faculty of Health Sciences, Calisia University, Kaszubska 13 Street, 62-800 Kalisz, Poland; jkamatysiak@gmail.com; 3 Department of Animal Physiology and Development, Faculty of Biology, Adam Mickiewicz University in Poznan, Uniwersytetu Poznańskiego 6 Street, 61-614 Poznań, Poland; rosin@amu.edu.pl; 4 Department of Innovative Biomaterials and Nanotechnologies, Institute of Natural Fibres and Medicinal Plants—National Research Institute, Wojska Polskiego 71B Street, 60-630 Poznań, Poland; elzbieta.kedzia@iwnirz.pl; 5 Department of Pediatric Gastroenterology and Metabolic Diseases, Poznan University of Medical Sciences, Szpitalna 27/33 Street, 60-572 Poznań, Poland; weronikazabek@wp.eu; 6 Department of Physiotherapy, Poznan University of Medical Sciences, 28 Czerwca 1956 r. 135/147 Street, 61-545 Poznań, Poland; jaroslaw.zawadzinski@gmail.com

**Keywords:** royal jelly, proteins, ProteoMiner^TM^, MALDI-TOF-MS, proteomics, beehive product

## Abstract

Royal jelly (RJ) is a complex, creamy secretion produced by the glands of worker bees. Due to its health-promoting properties, it is used by humans as a dietary supplement. However, RJ compounds are not fully characterized yet. Hence, in this research, we aimed to broaden the knowledge of the proteomic composition of fresh RJ. Water extracts of the samples were pre-treated using combinatorial hexapeptide ligand libraries (ProteoMiner^TM^ kit), trypsin-digested, and analyzed by a nanoLC-MALDI-TOF/TOF MS system. To check the ProteoMiner^TM^ performance in the MS-based protein identification, we also examined RJ extracts that were not prepared with the ProteoMiner^TM^ kit. We identified a total of 86 proteins taxonomically classified to *Apis* spp. (bees). Among them, 74 proteins were detected in RJ extracts pre-treated with ProteoMiner^TM^ kit, and only 50 proteins were found in extracts non-enriched with this technique. Ten of the identified features were hypothetical proteins whose existence has been predicted, but any experimental evidence proves their in vivo expression. Additionally, we detected four uncharacterized proteins of unknown functions. The results of this research indicate that the ProteoMiner^TM^ strategy improves proteomic identification in complex biological samples. Broadening the knowledge of RJ composition may contribute to the development of standards and regulations, enhancing the quality of RJ, and consequently, the safety of its supplementation.

## 1. Introduction

Bee products are unique, complex mixtures of biologically active compounds produced by honeybees (*Apis mellifera*). They exhibit strong health-promoting properties, appreciated since ancient times and used for medical purposes. Until now, they are recommended as drugs within the branch of alternative medicine called apitherapy [1]. One of the most sterling bee products, along with honey, bee pollen, and propolis, is royal jelly (RJ). It is a semi-liquid, milky white or yellowish secretion of worker bees’ salivary glands. Its sour-bitter-sweet taste (resulting from its acidic pH) and characteristic phenolic smell make it mostly recognizable among the so-called “superfoods” [2]. The term “superfoods” refers to foods that have beneficial effects on human health due to their rich nutrient content [3], and RJ fits this description well.

Chemically, fresh RJ consists of about 60–70% water, 11–23% carbohydrates, 9–18% proteins, 3–8% lipids, and small amounts of vitamins, minerals, free amino acids, and other constituents [2,4,5]. According to the literature reports, RJ is nonhomogeneous, displaying high variability between its main components [5,6,7,8,9,10]. Factors responsible for that variability are the metabolic and physiologic condition of worker bees and larval age [10,11,12], honeybee strain [13], place of origin, and year and season of the collection [11]. Since the fluctuations in RJ composition may significantly influence its biological activity, standardization, and quality control of RJ and other bee products seem to be an essential issue.

In a beehive, RJ serves as superior nourishment for all bee larvae until the prepupal stage. However, the honeybee queen is fed exclusively with RJ for its lifetime [14]. Since only the queen is able to reproduce, the epigenetic influence of RJ in the sexual maturation of the female bee larvae is unquestionable. Although it is not thoroughly investigated yet, how RJ provokes the queen’s development, monomeric major royal jelly protein 1 (MRJP1; royalactin), belonging to a major royal jelly proteins (MRJP) family, has been suggested to influence the larva differentiation into a bee queen [15]. Nevertheless, the mechanism of RJ’s action is probably more complicated, dependent not on the single compound but on the unique molecular blend found exclusively in RJ [16]. Hence, taking into account that RJ is the exceptional food of the bee queen, but it is also used in the treatment and prevention of some diseases, there is an urgent need to characterize the complete RJ composition, including macro- and micro-molecules, along with their biological functions. This will contribute to explaining the effects of royal jelly on the human body and also its beneficial and adverse properties in relation to both humans and animals.

Benefits arising from RJ consumption attract people to include it in their diet. However, the use of not thoroughly tested products poses a risk of side effects, such as inflammatory and allergic reactions, dermatitis, asthma, respiratory stress and bronchospasm, gastrointestinal problems, intestinal bleeding, and even death. Most of the factors responsible for both beneficial and harmful effects caused by RJ intake remain uncharacterized. Hence, taking into account that proteins are mostly accountable for the biological activity of the product, in this research, we aimed to broaden the knowledge of the proteomic composition of fresh RJ.

According to the available literature, several analytical methods have been proposed for the proteomic analysis of RJ. Since MRJPs are easily soluble in water, most of the researchers have extracted proteins contained in RJ using ultrapure water or buffer solutions [17,18,19,20,21,22]. Hence, in this study, we chose water as an optimal solvent. RJ is a complex product eminently rich in proteins [23]. Therefore, in other studies conducted worldwide, before identification, protein extracts were subjected to tryptic digestion and fractionated, using liquid chromatography or isoelectric fractionation approach. The identification analyses were performed mainly by electrophoretic techniques or mass spectrometry [17,18,19,20,21,24,25,26]. In this study, we decided to utilize proteolytic digestion, nanoLC (nano-liquid chromatography) and tandem MALDI-TOF/TOF (matrix-assisted laser desorption/ionization—time of flight/time of flight) mass spectrometer. 

In the proteomic investigation of natural products such as RJ, a major challenge is analysis of low-abundant proteins. Because there are significant differences in the concentrations of different proteins contained in RJ with a high content of MRJPs fraction, it is important to focus on proper samples preparation for proteomic analyses. However, according to the literature data, any previous study devised an efficient method for sub-proteome isolation. Therefore, we proposed the ProteoMiner^TM^ (Bio-Rad, Hercules, CA, USA) kit for proteins purifying and concentration. ProteoMiner^TM^ uses combinatorial hexapeptide ligand libraries, and it is a protein enrichment strategy allowing capturing low- and very low-abundance proteins. Such prepared RJ protein extracts were analyzed with advanced nanoLC-MALDI-TOF/TOF MS/MS (nano-liquid chromatography—matrix-assisted laser desorption/ionization—time of flight/time of flight mass spectrometry/mass spectrometry) technique. To the best of our knowledge, according to the available literature, it is the first attempt to analyse the proteomic fraction of RJ samples using ProteoMiner^TM^ protein enrichment approach.

## 2. Results

**Figure 1:**
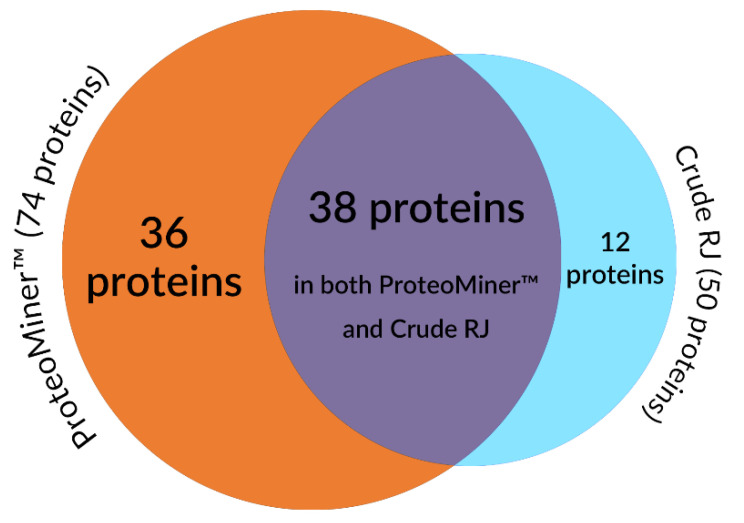
The number of proteins identified in unpurified samples, samples pre-treated with ProteoMiner^TM^, and both in unpurified and purified samples.

The methodology proposed for the analysis of RJ samples allowed us to identify a total of 86 proteins taxonomically classified to *Apis* spp. (Table 1). Among them, 50 proteins were detected in “crude” RJ water extracts, which were not pre-treated with ProteoMiner^TM^ kit, and 74 proteins were found in extracts enriched with this commercial technique (total in all fractions). The numbers of proteins identified in “crude” extracts, extracts pre-treated with ProteoMiner^TM^, and both in “crude” and ProteoMiner^TM^ pre-treated extracts are presented in Figure 1.

**Figure 2:**
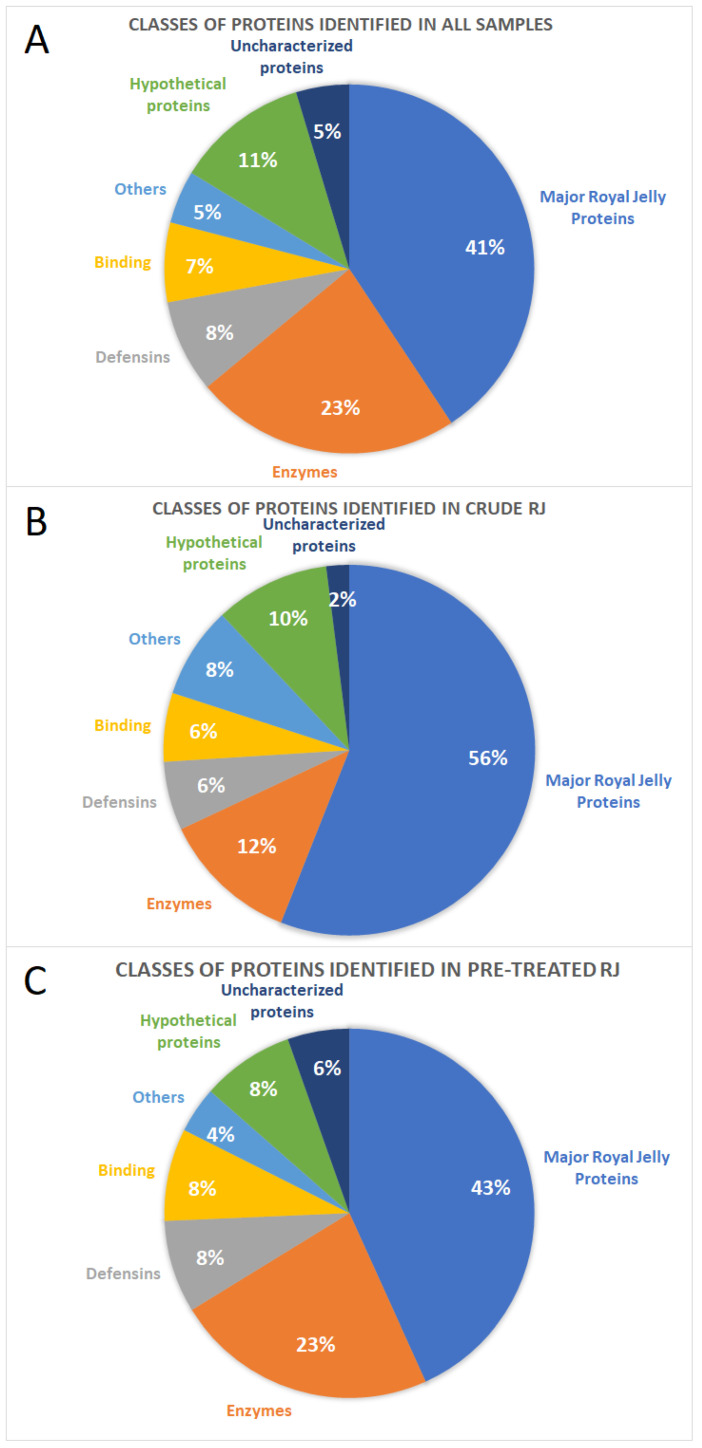
Classes of proteins identified (**A**) in total in all RJ “crude” and pre-treated; (**B**) only in crude (non-ProteoMiner^TM^ pre-treated) RJ samples; (**C**) only in ProteoMiner^TM^ pre-treated RJ samples.

In total, 35 proteins, accounting for 41% of all proteins identified in this study, belonged to the MRJPs family, responsible for honeybee nutrition and development (Figure 2A). Among them, fragments of MRJP1—MRJP7 and MRJP9 have been detected. The next important functional class of proteins were enzymes (20 proteins; 23% of all the identified proteins), with glucose dehydrogenase, alpha-glucosidase, venom acid phosphatase, lysozyme, and chymotrypsin inhibitor, among others. Into the most abundant protein classes, we also ranked defensins (7 proteins; representing 8% of all identified proteins), and binding proteins (6 proteins, accounting for 7% of all identified proteins), including regucalcin-like proteins. Additionally, four of all 86 proteins (5%) were classified into three other functional groups: remodeling proteins, snRNA processing proteins, and venom carbohydrate-rich proteins.

Moreover, ten identified proteins (11% of all identified proteins) were hypothetical proteins whose existence has been predicted, but there is not any experimental evidence proving their in vivo expression. Additionally, four uncharacterized proteins (5%) of unknown functions have been detected.

Proteins identified separately in extracts not pre-treated with ProteoMiner^TM^ enrichment technology were mainly MRJP (28 proteins out of 50, which represents 56% of proteins identified only in non-pretreated samples). The next functional classes of proteins included enzymes (6 identified proteins; 12%), defensins (3 proteins; 6%), binding proteins (3 proteins; 6%), and others (4 proteins; 8%). In these not-enriched samples, five hypothetical proteins (accounting for 10% of proteins identified in non-enriched samples) and one uncharacterized protein (2%) have been detected (Figure 2B).

Considering only extracts pre-treated with ProteoMiner^TM^ kit, the protein functional class distribution was comparable to overall results obtained from all compiled samples. MRJP (32 fragments) constituted 43% of all proteins identified in ProteoMiner^TM^ fractions. Besides, the most numerous classes were enzymes (17 proteins; 23% of proteins identified only in ProteoMiner^TM^ pre-treated samples), followed by defensins (6 proteins; 8%), binding proteins (6 proteins; 8%) and others (3 proteins; 4%). In those enriched samples, we also identified 6 (8% of all proteins identified in pre-treated fractions) hypothetical proteins and 4 (6%) uncharacterized proteins (Figure 2C).

Application of the ProteoMiner^TM^ technique resulted in expanded identification of proteins that belonged to almost all functional groups. Within the MRJPs group, 9 out of 35 (25.7%) MRJPs identified in total in this study were detected only in ProteoMiner^TM^ fractions. Moreover, 8 out of 19 (42.1%) enzymes identified in total were only detected in pre-treated samples. Regarding defensins, as many as 4 additional proteins belonging to this functional group were identified through the use of ProteoMiner^TM^, whereas only three defensins were identified in the unpurified samples (meaning that 57.1% of all identified defensins were detected using the ProteoMiner^TM^ technique). Similarly, out of 6 identified in total binding proteins, 3 of them (50%) were detected only in ProteoMiner^TM^ fractions. These results clearly indicate that the ProteoMiner^TM^ strategy significantly improves proteomic identification in complex biological samples, increasing the number of some identified functional proteins even more than twice. 

Moreover, compared to “crude” extracts, application of the ProteoMiner^TM^ kit enabled the detection of 5 more hypothetical proteins (out of 10 hypothetical proteins identified in total in this study) and 3 more uncharacterized proteins (out of 4 uncharacterized proteins identified in total in this study). These improvements in identification rate are especially beneficial when trying to find new proteomic features in bio-matrices represented by RJ.

## 3. Discussion

The goal of this study was to characterize the protein-peptide composition of fresh RJ using advanced nanoLC-MALDI-TOF/TOF MS/MS (nano-liquid chromatography—matrix-assisted laser desorption/ionization—time of flight mass spectrometry/mass spectrometry) technique. Despite its many unquestionable advantages, including versatility, ease of use, cost-effectiveness, and possibility to detect biomolecules in extremely low concentrations (even close to sub-femtomoles), the MALDI-TOF MS technique also has some limitations [27]. One of the most challenging issues is the detection of low-abundance proteins and peptides in very complex biological matrices. The presence of high concentration proteins and also other constituents, like lipids and salts, prevent direct analysis of all molecules contained in the sample [28]. Thus, proteins and peptides in low concentration ranges usually remain undetected. To deal with this problem, before MS analyses, biological samples must be purified, and their complexity and dynamic range need to be reduced. In this study, we applied a novel strategy based on a combinatorial hexapeptide ligand library (ProteoMiner^TM^). ProteoMiner^TM^ technique, by binding proteins to hexapeptides (through protein affinity interactions), resulted in reducing high-abundance proteins and enriching low-abundance proteins in RJ samples [29,30,31]. In this research, using the ProteoMiner^TM^ technique significantly improved the number of identified proteins, from 50 in non-enriched RJ water extracts to 74 in ProteoMiner^TM^ pre-treated extracts. 

Application of the proposed methodology resulted in the identification of a total number of 86 proteins (from all samples and ProteoMiner^TM^ fractions) taxonomically restricted to *Apis* spp. (*Apis mellifera*, *Apis cerana*, *Apis dorsata*, and *Apis florea*). As expected on the basis of available literature, the major royal jelly proteins family constituted the largest group. We identified 35 MRJPs or their precursors, which stands for 41% of the number of all identified proteins. Our results reflect previous independent proteomic studies, which reported that MRJPs account for up to 82–90% (*w*/*w*) of all proteins found in RJ [32,33,34]. It is also worth noting that in our study of only crude RJ, the percentage of MRJPs in relation to all identified proteins was as high as 56%. These results prove the utility of the ProteoMiner^TM^ enrichment technique in enhancing the protein identification ability by MS approaches.

MRJPs are a family of nine proteins, of which MRJP1, called apalbumin or royalactin, is the most abundant and also the first MRJP to be identified in 1992 [35]. It has been suggested that MRJPs have mainly nutritional and developmental functions. Moreover, MRJP1 has been reported to exhibit antibacterial and antiproliferative activity [14,36]. Interestingly, the effect of MRJPs and whole RJ on honeybee queen development is not only due to the nutritional properties. MRJP1, along with protein named apisimin, by specific complex formation, provide the optimal RJ viscosity. The viscosity is crucial for holding queen larvae on the surface of the comb cells, which are orientated vertically and open downwards [37,38]. The formation of MRJ1-apisimin polymer is strongly pH-dependent. Long structures are formed at pH about 4.0, measured in fresh RJ [37,39]. The specific acidity is sustained by the fatty acids content produced in the bees’ mandibular glands. 

The second most-abundant functional group of RJ-identified proteins in this study was enzymes. This group includes some important enzymes crucial for carbohydrate metabolisms, like glucose dehydrogenase, alpha-glucosidase and glucose oxidase. Those enzymes are suggested to play an essential role in facilitating the larvae’s digestion of sugars, which are found in RJ in large quantities [19]. The next relevant enzyme identified in our study was lysozyme isoform 1 and 2. Lysozyme is a protein commonly found in animals. It is a natural component of various secretions such as tears, saliva, mucus and milk. Lysozyme exhibits a strong antimicrobial activity, which in beehive prevents bacterial infection and disease [40,41]. Furthermore, because of their human benefits, the antibacterial activities of RJ are worthy of notice. These properties were reported as far back as 1939 [42]. According to the literature, besides the lysozyme, a 5.5 kDa defensin peptide named royalisin, and 10-hydroxy-2-decenoic acid (10-HDA) are mainly responsible for the antibacterial activity of RJ [43,44,45]. Antimicrobial properties, along with anti-inflammatory, immunomodulatory, antioxidant, moisturising, toning, and anti-ageing activities, make RJ a perfect dermatological product. Hence, RJ is used for treating various skin lesions and wounds [46,47]. Antibacterial defensins, including royalisin preproprotein (named defensin-1 preproprotein), were also identified in this study.

However, among the enzymes included in RJ, we found venom acid phosphatase Acph-1-like and venom serine protease 34. These proteins, along with MRJP8, MRJP9 and icarapin variant 2 precursor (also found in this study), are classified as bee venom allergens (based on Allergen Nomenclature Database, www.allergen.org, accessed on 8 March 2021). Allergens are responsible for the occurrence of allergic reactions in humans and animals after exposition to bee products [48,49,50]. The highest risk of an allergic reaction, both local or systemic, is associated with exposure to *Hymenoptera* venom, especially after the insect sting. Since allergens are present also in other bee products, like RJ (what was proven in this study), bee products should be consumed with caution and in moderation. 

According to literature data, allergy to royal jelly is most common IgE-mediated, but contact allergic reactions (contact dermatitis) are also possible. IgE allergic reactions to royal jelly cause symptoms such as urticaria, angioedema, eczema, allergy rhinitis, conjunctivitis, bronchospasm or even anaphylactic shock [51,52,53]. Patients with bronchial asthma and atopic dermatitis are particularly predisposed to severe reactions to RJ. Even 17% adult patient with asthma [54] and one third of patient with atopic dermatitis [55] have specific IgE to RJ. Reported cases of allergy to royal jelly concern both people who have consumed this product for the first time or after long use. Allergy reaction to RJ after the first use is possible because of cross-reactivity between RJ and other allergens. Cross-reactions between RJ and bee venom can be associated with proteins found in our study—venom acid phosphatase Acph-1-like, venom serine protease 34, MRJP8, MRJP9 and icarapin variant 2 precursor. Moreover, cross-reactions are also possible with very typical allergens like house dust mite (*Dermatophagoides farinae*, *Dermatophagoides pteronyssinus*), German cockroach or crab [55]. In conclusion, RJ can cause an allergic reaction because it contains allergenic proteins and, further, because of cross-reactions with typical allergens. Patients with venom allergy, asthma, atopic dermatitis, and allergic rhinitis should be careful before consuming RJ.

Additionally, to minimalize the risk of dangerous adverse effects, bee products should be thoroughly tested for allergens, both qualitatively and quantitatively. However, despite its undeniable beneficial pro-health properties, the use of RJ in people with local and/or systemic *Hymenoptera* venom allergic reactions should be avoided.

Although in this study we focused mainly on nutritive MRJPs, and enzymes, defensins, and allergens, we also identified other functional groups of proteins included binding proteins, chymotrypsin inhibitors, protein SERAC1-like, and integrator complex subunit 7-like isoform 1. Those proteins show mainly regulatory activities.

The most interesting group of the identified biomolecules, regarding the possibility of learning about new properties and functions of royal jelly, are hypothetical and uncharacterized proteins. In this study, we detected 10 hypothetical proteins whose existence has been predicted based on the genetic information, but there is any experimental evidence proving their in vivo expression. The functions of these proteins, as in the case of uncharacterized proteins, have not been investigated yet. For these proteins, we performed BLAST analysis (https://blast.ncbi.nlm.nih.gov/, accessed on 11 January 2021) to find similar regions in various biological sequences. This approach helps to assess functional relationships between proteins and identify members of protein families. Results of our analysis are presented in Table 2. 

For the identified gi|110756431 protein (Predicted: hypothetical protein LOC725074 [*Apis mellifera*]), we found a significant alignment sequence referred to omega-conotoxin-like protein 1 [*Apis mellifera*]. This protein was also found in bee venom in our previous studies, based on HPLC, nanoLC-MALDI-TOF/TOF MS/MS, and LC–ESI–QToF methodology [56,57]. With a high degree of probability, we can conclude that the molecule we have identified corresponds entirely to the results obtained from the BLAST platform, as both query coverage and per cent identity equal 100%. It means that our identified sequence exactly corresponds to conotoxin-like protein 1. As far as we know, this protein has never been reported to be included in RJ before. Detection of conotoxin-like protein 1 in royal jelly is a particularly important discovery in terms of the safety of RJ and also its potential therapeutic use. Conotoxins are small, disulfide-rich, neurotoxic peptides isolated from the marine cone snails venom. There are many types of these molecules, differing in structure and function [58]. The mechanism of their action has not been utterly elucidated yet. However, it is known that they bind to ion channels, neurotransmitters, transporters, and neural receptors [59]. Because of their selective binding to neuronal targets, conotoxins may be used for the developments of new drugs. Moreover, these peptides may be potentially used in the treatment of neurodegenerative conditions, like Alzheimer or Parkinson diseases [60]. Results of our study suggest that conotoxin may also affect the therapeutic effect of RJ, especially since some studies revealed that RJ shows a neuroprotective effect [61,62,63]. What is interesting, is that it has been investigated whether the consumption of RJ can be beneficial in the treatment of behavioral deficits in Alzheimer’s disease in rabbits [64]. Thus, the presence of conotoxin-like protein 1 in RJ may contribute to elucidate the neuroprotective effect caused by RJ. Interestingly, Kaplan et al. [65] found this protein to be mainly expressed in the bee brain. It’s also worth noting that expression of a protein with an approximate mass of 8.2 kDa, referred to as omega-conotoxin-like protein 1, has been reported by Gätschenberger et al. [66] to be induced in the haemolymph of young drones as a response to a septic injury. It may suggest that this protein participates in an immune response. 

The next protein, for which the sequence similarity was noticed, was ataxin-2. This protein has been found in the BLAST analysis of gi|380026601 (Predicted: uncharacterised protein LOC100863702 [*Apis florea*]). However, database searching returned the BLAST scores under 100% (77% query coverage and 88.41% per cent identity). Therefore, the presence of this protein (or its homolog) in royal jelly should be confirmed in further research. Ataxin-2 is an RNA-binding protein, which regulates mRNA stability and translation. It participates in cell death, calcium homeostasis, and cellular metabolism [67,68]. In insects, ataxin-2 is involved in the regulation of circadian rhythms but also in the development of peripheral tissue [69,70,71]. This may explain the presence of ataxin-2 in RJ. 

Other peptides that have been found to be similar to the sequences detected in RJ are odorant receptor Or1 and chymotrypsin inhibitor. These proteins play mainly regulatory functions. Odorant receptor Or1 targets in bee’s periphery nervous system. It participates in the odorants transport and binding, and therefore, in the behavioral response to specific odorants [72]. Chymotrypsin inhibitor prevents proteolysis of proteins present in RJ. For the chymotrypsin inhibitor, 100% similarity to gi|328783471 (Predicted: hypothetical protein LOC725114 isoform 1 [*Apis mellifera*]) was found. However, query coverage was lower (84%) for the analysis of gi|328783362 (Predicted: hypothetical protein LOC725249 [*Apis mellifera*]). Regarding odorant receptor Or1, the similarity with Predicted: uncharacterised protein LOC100864410 [*Apis florea*] detected in our study was expressed with 98% query coverage and 89.68% per cent identity. Therefore, these proteomic features require further research.

Other peptides for which a partial similarity to the RJ-detected proteins was found were Chorion peroxidase, regucalcin, kielin/chordin-like protein, and low-density lipoprotein receptor 1-like isoform X3. All those proteins bear mainly regulatory functions. However, due to lower BLAST scores (see Table 2), a certain identification must be proved by additional analyses. For the remaining hypothetical and uncharacterized proteins found in royal jelly, no defined references were found in BLAST analysis. The structure, functions and properties of these proteins should be investigated in future research. However, gi|380012917 (Predicted: uncharacterized protein LOC100870850 [*Apis florea*]), and gi|48094573 (uncharacterized protein LOC408608 [*Apis mellifera*]) were found in *Hymenoptera* venom in our previous study [56].

As the number of fully sequenced honeybee proteins and peptides increases, omics and bioinformatics technologies provide a tremendous amount of information about sequential biomolecules that function in various biological aspects of the honeybee and that are found in its products. In the postgenomic era, the international project i5K (http://i5k.github.io/.2018, accessed on 16 February 2021) not only played an important role in understanding the honeybee genome but also resulted in extensive research on the bee’s proteome and the identification of many active proteins and peptides in its products. For a number of new substances produced by honeybee, only one type of biological activity has been identified, and their wider physiological role is unknown. Because many substances often exhibit pleiotropic biological activity, there is a need to understand a wider spectrum of biological activity for newly discovered compounds. It is also necessary to develop new, specific, and sensitive physiological, pharmacological, or toxicological bioassays. In the last decade, we have been observing new trends in the development of modern biotechnology, where besides medical, industrial, food and plant protection biotechnology, the term “Yellow Biotechnology” was introduced as an alternative term for insect biotechnology, opening new horizons for multidisciplinary research in the field of experimental entomology [73]. Yellow biotechnology is defined as the use of biotechnology to transform insects, their molecules, cells or organs into products and services with great potential for applications in biomedicine, pharmacy, and industry, where the honeybee may be one of the candidates.

## 4. Materials and Methods

### 4.1. Royal Jelly

In this study, we analyzed three samples of fresh RJ, collected in June 2019. The non-commercial apiary, from which all fresh samples were collected, is located in Góry Złotnickie village (51°87′504″ N, 18°12′431″ E) in Poland (Greater Poland Voivodeship, west-central Poland). Right after the collection, samples were stored at −80 °C until analyses. All RJ samples were analyzed in three technical replicates.

### 4.2. Pre-Treatment of the Royal Jelly Samples

Samples of fresh royal jelly were first suspended in ultrapure water in a concentration of 330 mg/mL. The RJ-water suspensions were mixed with vortex for 1 min and then sonicated for 20 min. After sonication, suspensions were vortex-mixed for 1 min and subsequently spun with 13,000 RPM (9600 RCF) for 20 min. Collected supernatants (in this study called “extracts”) were used in further steps.

### 4.3. Relative Protein Enrichment—ProteoMiner^TM^ Hexapeptide Ligand Library

For the presented research, we applied a commercial ProteoMiner^TM^ Sequential Elution Small Capacity Kit (Bio-Rad, Hercules, CA, USA). All enrichment steps were performed strictly according to the manufacturer’s instructions. The only difference from the manufacturer’s protocol was the type of the sample solution loaded onto the ProteoMiner^TM^ column. Instead of the 0.2 mL of serum (for which the protocol is optimized), we added 0.2 mL of RJ extract, prepared as described above (see Section 4.2). In brief, 0.2 mL of the RJ water extracts were loaded onto pre-conditioned ProteoMiner^TM^ columns containing slurry beads and incubated at room temperature (RT) on a platform shaker. Next the samples were washed with phosphate buffer saline (PBS), and sequentially eluted with solvents: 1 M sodium chloride, 20 mM HEPES, pH 7.4 (Elution Reagent 1), 200 mM glycine, pH 2.4 (Elution Reagent 2), 60% ethylene glycol in water (Elution Reagent 3), and 33.3% 2-propanol, 16.7% acetonitrile (ACN), 0.1% trifluoroacetic acid (TFA) (Elution Reagent 4). In total, 4 separate fractions were obtained from each sample. These fractions were further trypsin digested and then purified with ZipTip.

### 4.4. Trypsin Proteolytic Digestion and ZipTip Concentration and Purification

All RJ fractions derived from the ProteoMiner^TM^ enrichment method and additionally crude RJ solutions (water suspended and sonicated, but not fractionated with ProteoMiner^TM^; three technical repetitions) were subjected to digestion with trypsin (Promega, Madison, WI, USA). We applied a modified protocol from pierce in-solution tryptic digestion kit. In brief, before adding trypsin, proteins in extracts (both ProteoMiner^TM^ fractions and “crude” extracts non-enriched with ProteoMiner^TM^) were denatured, reduced, and alkylated. Subsequently, after trypsin addition, RJ extracts were digested at 37 °C overnight. In the next step, ZipTip C18 (Millipore, Bedford, MA, USA) reverse phase chromatography micropipette tips were used to purify, desalt and concentrate digested RJ protein extracts before mass spectrometry analyses. Tips were conditioned using ACN and 0.1% TFA/water. Then, each solution of the digested proteins extracted from the RJ samples (the exact solutions formed due to trypsin digestion of the RJ extracts, without any additional reagents) was loaded onto ZipTip tips according to the manufacturer’s instruction. Sample solutions were aspirated and dispensed in 10 cycles for maximum binding of proteins. After washing with 0.1% TFA, bound proteins and peptides were eluted with 50% ACN in 0.1% TFA.

### 4.5. NanoLC-MALDI-TOF/TOF MS/MS Analyses

For the identification of peptides and proteins included in RJ, the nanoLC-MALDI-TOF/TOF MS/MS technique was applied. Subsequent to ZipTip depletion, RJ protein extracts were subjected to nanoLC fractionation. The nanoLC set consisted of EASY-nLC II (Bruker Daltonics, Bremen, Germany) nanoflow HPLC system, and Proteineer-fc II (Bruker Daltonics, Bremen, Germany) collector of fractions. The nanoLC system parts were: NS-MP-10 BioSphere C18 (NanoSeparations, Nieuwkoop, the Netherlands) trap column (20 mm × 100 µm I.D., particle size 5 µm, pore size 120 Å), and an Acclaim PepMap 100 (Thermo Scientific, Sunnyvale, CA, USA) column (150 mm × 75 µm I.D., particle size 3 µm, pore size 100 Å). The gradient elution method was set on 2–50% of ACN in 96 min (mobile phase A—0.05% TFA in water, mobile phase B—0.05% TFA in 90% ACN). The flow rate for separation was 300 nL/min, and the volume of the sample eluent injected into the chromatography column was 4 µL. The nanoLC system was operated by HyStar 3.2 (Bruker Daltonics, Bremen, Germany) software. In total, 384 fractions were obtained in the nanoLC separation process. Fractions were mixed with a matrix solution consisted of 36 µL of HCCA saturated solution in 0.1% TFA and acetonitrile (90:10 *v*/*v*), 748 µL of acetonitrile and 0.1% TFA (95:5 *v*/*v*) mixture, 8 µL of 10% TFA, and 8 µL of 100 mM ammonium phosphate, and spotted automatically onto the AnchorChip 384 (Bruker Daltonics, Bremen, Germany) target plate by the collector of fractions. Fractions were subsequently subjected to MS/MS analysis. For this purpose, UltrafleXtreme (Bruker Daltonics, Bremen, Germany) mass spectrometer was used (mass range *m*/*z* of 700–3500, reflectron mode). External calibration was performed with a mixture of Peptide Calibration Standard (Bruker Daltonics, Bremen, Germany). The list of the precursor ions for the identification was established with WARP-LC (Bruker Daltonics, Bremen, Germany) software. For the spectra acquisition, processing and evaluation FlexControl 3.4, FlexAnalysis 3.4 and BioTools 3.2 (Bruker Daltonics, Bremen, Germany) software were used. For the identification of discriminative proteins and peptides, an NCBI database and Mascot 2.4.1 search engine with taxonomic restriction to *Apis* spp. were applied. The protein search parameters were as follows: fragment ion mass tolerance *m*/*z* ± 0.7, precursor ion mass tolerance ±50 ppm, peptide charge +1, monoisotopic mass.

## 5. Conclusions

The functional and health-promoting properties of RJ make it one of the most attractive healthy ingredients. However, because of an overly complicated composition, RJ is not yet fully characterized and may cause adverse effects. Therefore, in this study, we proposed an advanced strategy based on combinatorial hexapeptide ligand libraries (ProteoMiner^TM^ kit) for the proteomic analysis of RJ water extracts. We identified a total of 86 proteins taxonomically classified to *Apis* spp. (bees). In addition to the well-known components of RJ, we identified 10 hypothetical proteins and 4 uncharacterized proteins. We also proved the utility of the ProteoMiner^TM^ technique in the analysis of complex biological samples, as 74 out of all identified proteins were detected in RJ extracts pre-treated with ProteoMiner^TM^ kit, and only 50 proteins were found in extracts that were not enriched with this technique. Broadening the knowledge of RJ composition may contribute to the development of standards and regulations, enhancing the quality of RJ, and consequently, the safety of its supplementation. Moreover, better characterization of proteins and peptides found in RJ may support the understanding of the functional properties of RJ regarding both humans and bees.

## Figures and Tables

**Table 1:** List of proteins identified in royal jelly found in: CRJ—“crude” (non-enriched with ProteoMiner^TM^ technique) water extracts of royal jelly; F1, F2, F3, F4—separate fractions obtained from ProteoMiner^TM^ elution with 1 M sodium chloride, 20 mM HEPES, pH 7.4 (F1), 200 mM glycine, pH 2.4 (F2), 60% ethylene glycol in water (F3), and 33.3% 2-propanol, 16.7% acetonitrile (ACN), 0.1% trifluoroacetic acid (TFA) (F4); MW—molecular weight; pI—isoelectric point. Fractions, in which proteins were detected, marked with “x”.

Accession	Protein Name	Function	MW [kDa]	pI	CRJ	F1	F2	F3	F4
gi|110749126	Predicted: glucose dehydrogenase [acceptor] isoform 3 [*Apis mellifera*]	Enzyme	70.1	6.7		x			
gi|110751029	Predicted: e3 ubiquitin-protein ligase IAP-3-like [*Apis mellifera*]	Enzyme	43.2	6.4					x
gi|110756431	Predicted: hypothetical protein LOC725074 [*Apis mellifera*]		8.3	10.2	x				
gi|110758964	Predicted: regucalcin-like [*Apis mellifera*]	Binding	10.2	9.4	x				x
gi|110763647	Predicted: hypothetical protein LOC726323 [*Apis mellifera*]		18.5	7.9					x
gi|13184963	defensin [*Apis mellifera*]	Defense	6.3	6.5					x
gi|166795901	apolipophorin-III-like protein precursor [*Apis mellifera*]	Binding	21.3	5.4	x	x	x	x	x
gi|202078658	defensin [*Apis cerana cerana*]	Defense	10.6	7.8	x				
gi|202078660	defensin [*Apis cerana cerana*]	Defense	10.7	7.8			x		
gi|254548151	defensin precursor [*Apis cerana*]	Defense	8.7	6.4	x	x	x	x	x
gi|254548155	defensin precursor [*Apis mellifera*]	Defense	8.8	5.9	x	x	x	x	x
gi|254910938	defensin-1 preproprotein [*Apis mellifera*]	Defense	10.7	6.4			x		
gi|258678306	MRJP9 [*Apis cerana*]	Honeybee nutrition and development	19.4	9.2			x		
gi|258678310	MRJP5 [*Apis dorsata*]	Honeybee nutrition and development	21.3	4.7	x				
gi|258678314	MRJP6 [*Apis florea*]	Honeybee nutrition and development	21.5	4.4		x	x		
gi|258678316	MRJP9 [*Apis florea*]	Honeybee nutrition and development	21.0	5.5	x	x	x	x	x
gi|283105164	alpha-glucosidase III [*Apis dorsata*]	Enzyme	65.5	5.0	x				x
gi|284182838	major royal jelly protein 4 [*Apis mellifera*]	Honeybee nutrition and development	53.0	5.9	x	x	x	x	
gi|284812514	MRJP5 [*Apis mellifera*]	Honeybee nutrition and development	70.1	6.1	x	x	x	x	x
gi|288872651	major royal jelly protein [*Apis mellifera*]	Honeybee nutrition and development	61.6	6.7	x	x	x	x	x
gi|28972896	major royal jelly protein-like protein [*Apis dorsata*]	Honeybee nutrition and development	9.2	9.8	x	x	x	x	x
gi|328775853	Predicted: DNA replication licensing factor MCM4-like [*Apis mellifera*]	Binding	80.7	6.9		x			
gi|328777366	Predicted: hypothetical protein LOC100577348 [*Apis mellifera*]		61.1	10.5			x		
gi|328779534	Predicted: hypothetical protein LOC552041 [*Apis mellifera*]		79.3	4.2					x
gi|328782858	Predicted: hypothetical protein LOC410515 [*Apis mellifera*]		190.2	6.4	x				
gi|328783362	Predicted: hypothetical protein LOC725249 [*Apis mellifera*]		6.3	4.9	x				
gi|328783471	Predicted: hypothetical protein LOC725114 isoform 1 [*Apis mellifera*]		10.1	5.7	x				
gi|328784821	Predicted: hypothetical protein LOC100577210 [*Apis mellifera*]		11.6	5.9	x	x		x	x
gi|328789019	Predicted: protein SERAC1-like [*Apis mellifera*]	Remodeling	87.5	9.2		x			
gi|328790726	Predicted: venom acid phosphatase Acph-1-like [*Apis mellifera*]	Enzyme	42.6	8.5					x
gi|328792767	Predicted: hypothetical protein LOC724993 [*Apis mellifera*]		43.8	5.4			x		
gi|328793775	Predicted: s-adenosylmethionine decarboxylase proenzyme-like, partial [*Apis mellifera*]	Enzyme	32.1	4.6	x				
gi|328794347	Predicted: major royal jelly protein 3-like, partial [*Apis mellifera*]	Honeybee nutrition and development	42.7	6.5	x		x		
gi|33358394	major royal jelly protein MRJP1 [*Apis cerana cerana*]	Honeybee nutrition and development	49.0	5.4	x	x	x	x	x
gi|380011960	Predicted: slit homolog 2 protein-like [*Apis florea*]	Binding	155.3	6.0		x			
gi|380012917	Predicted: uncharacterized protein LOC100870850 [*Apis florea*]		18.9	7.2				x	
gi|380013532	Predicted: Low Quality Protein: clathrin heavy chain-like [*Apis florea*]	Binding	187.7	5.6			x		
gi|380016522	Predicted: probable bifunctional methylenetetrahydrofolate dehydrogenase/cyclohydrolase 2-like [*Apis florea*]	Enzyme	39.9	9.6		x			
gi|380017034	Predicted: glucosylceramidase-like [*Apis florea*]	Enzyme	60.8	5.5				x	x
gi|380019073	Predicted: lysozyme 1-like isoform 2 [*Apis florea*]	Enzyme	13.8	4.6	x			x	x
gi|380020436	Predicted: regucalcin-like [*Apis florea*]	Binding	37.7	5.3	x	x		x	x
gi|380022658	Predicted: major royal jelly protein 3-like [*Apis florea*]	Honeybee nutrition and development	62.7	7.8	x	x			
gi|380022660	Predicted: major royal jelly protein 4-like [*Apis florea*]	Honeybee nutrition and development	56.1	6.1				x	
gi|380022665	Predicted: major royal jelly protein 1-like [*Apis florea*]	Honeybee nutrition and development	43.9	5.3	x	x	x	x	x
gi|380022667	Predicted: major royal jelly protein 2-like [*Apis florea*]	Honeybee nutrition and development	49.1	5.7	x	x			
gi|380022669	Predicted: major royal jelly protein 2-like [*Apis florea*]	Honeybee nutrition and development	49.3	6.0	x	x	x	x	x
gi|380022673	Predicted: major royal jelly protein 5-like [*Apis florea*]	Honeybee nutrition and development	34.2	4.7			x		
gi|380022681	Predicted: major royal jelly protein 5-like isoform 2 [*Apis florea*]	Honeybee nutrition and development	47.5	9.2		x			
gi|380023404	Predicted: uncharacterized protein LOC100869599 [*Apis florea*]		18.7	9.5	x			x	x
gi|380024584	Predicted: chymotrypsin inhibitor-like [*Apis florea*]	Enzyme inhibitor	5.6	10.0	x				
gi|380024588	Predicted: chymotrypsin inhibitor-like [*Apis florea*]	Enzyme inhibitor	8.0	5.0	x				
gi|380025248	Predicted: alkylated DNA repair protein alkB homolog 8-like [*Apis florea*]	Enzyme	68.2	9.3	x	x			
gi|380025500	Predicted: venom acid phosphatase Acph-1-like [*Apis florea*]	Enzyme	39.9	4.9					x
gi|380025661	Predicted: glucose dehydrogenase [acceptor]-like [*Apis florea*]	Enzyme	67.9	6.4	x	x		x	x
gi|380026601	Predicted: uncharacterized protein LOC100863702 [*Apis florea*]		9.9	5.0				x	x
gi|380027252	Predicted: uncharacterized protein LOC100864410 [*Apis florea*]		33.4	9.7			x		
gi|380028593	Predicted: Low Quality Protein: delta-1-pyrroline-5-carboxylate synthase-like [*Apis florea*]	Enzyme	84.9	9.1			x		
gi|40218299	major royal jelly protein MRJP5 [*Apis cerana cerana*]	Honeybee nutrition and development	70.5	8.8		x			
gi|40218301	major royal jelly protein MRJP2 [*Apis cerana cerana*]	Honeybee nutrition and development	53.0	9.1	x		x		x
gi|40557703	major royal jelly protein MRJP1 precursor [*Apis cerana*]	Honeybee nutrition and development	48.9	5.4	x	x	x	x	x
gi|40557705	major royal jelly protein MRJP2 precursor [*Apis cerana*]	Honeybee nutrition and development	52.5	8.9	x	x	x	x	x
gi|42601246	major royal jelly protein MRJP5 precursor [*Apis cerana*]	Honeybee nutrition and development	68.2	9.3		x			x
gi|46358503	major royal jelly protein 2 [*Apis cerana*]	Honeybee nutrition and development	52.4	8.9	x	x	x	x	x
gi|48094573	Predicted: hypothetical protein LOC408608 [*Apis mellifera*]		19.4	7.5				x	x
gi|48101366	Predicted: venom serine protease 34 [*Apis mellifera*]	Enzyme	44.6	5.9				x	
gi|562090	defensin precursor [*Apis mellifera*]	Defense	10.7	6.4			x	x	
gi|56422035	major royal jelly protein 3 [*Apis mellifera carnica*]	Honeybee nutrition and development	65.7	7.1	x	x	x	x	x
gi|56422037	major royal jelly protein 3 [*Apis cerana*]	Honeybee nutrition and development	69.2	9.3	x	x		x	
gi|56422041	major royal jelly protein 3 [*Apis florea*]	Honeybee nutrition and development	59.3	6.4	x				
gi|57546160	major rojal jelly protein 7 [*Apis cerana*]	Honeybee nutrition and development	24.9	5.5	x	x	x	x	x
gi|58585090	glucose oxidase [*Apis mellifera*]	Enzyme	67.9	6.5				x	x
gi|58585098	major royal jelly protein 1 precursor [*Apis mellifera*]	Honeybee nutrition and development	48.9	5.0	x	x	x	x	x
gi|58585108	major royal jelly protein 2 precursor [*Apis mellifera*]	Honeybee nutrition and development	51.0	7.0	x	x	x	x	x
gi|58585138	major royal jelly protein 5 precursor [*Apis mellifera*]	Honeybee nutrition and development	70.2	5.9	x				
gi|58585142	major royal jelly protein 3 precursor [*Apis mellifera*]	Honeybee nutrition and development	61.6	6.5	x		x		x
gi|58585164	alpha-glucosidase precursor [*Apis mellifera*]	Enzyme	65.5	4.9					x
gi|58585170	major royal jelly protein 4 precursor [*Apis mellifera*]	Honeybee nutrition and development	52.9	5.9	x	x	x	x	x
gi|58585188	major royal jelly protein 6 precursor [*Apis mellifera*]	Honeybee nutrition and development	49.8	5.9	x	x	x	x	x
gi|60115688	icarapin-like precursor [*Apis mellifera*]	Venom carbohydrate-rich protein	24.8	4.4	x	x		x	x
gi|62198227	major royal jelly protein 7 precursor [*Apis mellifera*]	Honeybee nutrition and development	50.5	4.8	x	x	x	x	x
gi|66504790	Predicted: integrator complex subunit 7-like isoform 1 [*Apis mellifera*]	snRNA processing	105.9	9.5	x				
gi|66511554	Predicted: glucosylceramidase-like isoform 1 [*Apis mellifera*]	Enzyme	59.3	5.2	x	x	x	x	x
gi|66564326	Predicted: plasma glutamate carboxypeptidase-like isoform 1 [*Apis mellifera*]	Enzyme	52.9	5.1					x
gi|66565246	Predicted: lysozyme isoform 1 [*Apis mellifera*]	Enzyme	17.1	4.6				x	x
gi|76496465	major royal jelly protein 3 [*Apis dorsata*]	Honeybee nutrition and development	66.9	6.6	x	x	x	x	x
gi|94471624	icarapin variant 2 precursor [*Apis mellifera*]	Venom carbohydrate-rich protein	19.6	4.2		x			

**Table 2:** Results of BLAST analysis.

Accession	Protein Name	Significant Alignment Sequence	Query Coverage	Percent Identity	E-Value
gi|110756431	Predicted: hypothetical protein LOC725074 [*Apis mellifera*]	omega-conotoxin-like protein 1 [*Apis mellifera*]	100%	100.00%	2.00 × 10^−47^
gi|110763647	Predicted: hypothetical protein LOC726323 [*Apis mellifera*]	uncharacterized protein LOC726323 isoform X1 [*Apis mellifera*]	100%	99.39%	1.00 × 10^−116^
gi|328777366	Predicted: hypothetical protein LOC100577348 [*Apis mellifera*]	uncharacterized protein LOC100577348 isoform X2 [*Apis mellifera*]	98%	98.37%	0.0
gi|328779534	Predicted: hypothetical protein LOC552041 [*Apis mellifera*]	uncharacterized protein LOC102655185 isoform X1 [*Apis mellifera*]	100%	71.55%	5.00 × 10^−92^
gi|328782858	Predicted: hypothetical protein LOC410515 [*Apis mellifera*]	uncharacterized protein LOC410515 [*Apis mellifera*]	93%	100.00%	0.0
		chorion peroxidase [*Habropoda laboriosa*]	93%	62.86%	0.0
gi|328783362	Predicted: hypothetical protein LOC725249 [*Apis mellifera*]	chymotrypsin inhibitor-like [*Apis mellifera*]	84%	100.00%	1.00 × 10^−25^
gi|328783471	Predicted: hypothetical protein LOC725114 isoform 1 [*Apis mellifera*]	chymotrypsin inhibitor [*Apis mellifera*]	100%	100.00%	2.00 × 10^−59^
gi|328784821	Predicted: hypothetical protein LOC100577210 [*Apis mellifera*]	uncharacterized protein LOC413627 [*Apis mellifera*]	77%	100.00%	6.00 × 10^−45^
		regucalcin [*Apis cerana cerana*]	77%	94.81%	4.00 × 10^−45^
gi|328792767	Predicted: hypothetical protein LOC724993 [*Apis mellifera*]	uncharacterized protein LOC724993 [*Apis mellifera*]	83%	90.37%	0.00 × 10^+00^
		Predicted: kielin/chordin-like protein [*Trachymyrmex zeteki*]	82%	45.91%	2.00 × 10^−85^
		kielin/chordin-like protein [*Nasonia vitripennis*]	81%	47.06%	3.00 × 10^−89^
gi|380012917	Predicted: uncharacterized protein LOC100870850 [*Apis florea*]	uncharacterized protein LOC408608 [*Apis mellifera*]	99%	73.33%	2.00 × 10^−84^
gi|380023404	Predicted: uncharacterized protein LOC100869599 [*Apis florea*]	uncharacterized protein LOC102654257 [*Apis mellifera*]	100%	94.71%	7.00 × 10^−116^
		low-density lipoprotein receptor 1-like isoform X3 [*Vespa mandarinia*]	98%	55.88%	7.00 × 10^−57^
gi|380026601	Predicted: uncharacterized protein LOC100863702 [*Apis florea*]	ataxin-2 [*Apis cerana cerana*]	77%	88.41%	5.00 × 10^−30^
gi|380027252	Predicted: uncharacterized protein LOC100864410 [*Apis florea*]	odorant receptor Or1 [*Apis mellifera*]	98%	89.68%	0.0
gi|48094573	Predicted: hypothetical protein LOC408608 [*Apis mellifera*]	uncharacterized protein LOC408608 [*Apis mellifera*]	100%	100.00%	3.00 × 10^−124^

## Data Availability

The data presented in this study are contained within the article.
